# Increased functional and structural skin capillary density in type 1 diabetes patients with vascular complications

**DOI:** 10.1186/1758-5996-1-24

**Published:** 2009-12-03

**Authors:** Eduardo Tibiriçá, Elba Rodrigues, Roberta Cobas, Marilia B Gomes

**Affiliations:** 1 Department of Medicine, Diabetes Unit, State University of Rio de Janeiro, Rio de Janeiro, Brazil; 2 Laboratory of Cardiovascular Investigation, Oswaldo Cruz Institute, Rio de Janeiro, Rio de Janeiro, Brazil

## Abstract

The present study was designed to study skin capillary density and recruitment of the upper and lower extremities in patients with type 1 diabetes with vascular complications, when compared to patients without complications and healthy subjects. We used intravital video-microscopy to measure basal and maximal (during venous congestion) skin capillary densities as well as capillary recruitment using post-occlusive reactive hyperemia (PORH) in the dorsum of the fingers and toes.

Our results showed that besides microvascular vasodilation and loss of autoregulatory capacity, patients with type 1 diabetes with vascular complications present increased skin capillary density in both extremities.

## 

Diabetes is one of the major risk factors for development of micro- and macrovascular disorders. Microangiopathy in type 1 diabetes is characterized by a generalized precapillary vasodilation and increased microvascular blood flow [1], resulting in capillary hypertension and endothelial dysfunction, which in turn lead to vascular complications [2]. Using intravital videomicroscopy, we recently demonstrated that capillary recruitment is impaired in patients with type 1 diabetes who do not display clinical complications [3]. The present study was designed to study skin capillary density and recruitment in patients with type 1 diabetes with vascular complications.

## Methods

This cross-sectional observational study was performed on a group of 88 outpatients consecutively attending the diabetes clinic of the State University of Rio de Janeiro and also included 47 control subjects without familiar history for diabetes and without diabetes (fasting blood glucose < 100 mg/dl), matched for age, gender and smoking habit (Table 1). The inclusion criteria were patients with diabetes diagnosed before 30 years of age who had only used insulin since the diagnosis, without symptoms of diabetes decompensation or acute infection. In females, all measurements were performed during the follicular phase of the menstrual cycle. Among diabetic patients, 26 (29.5%) had complications (microalbuminuria, retinopathy or coronary artery disease). The study was approved by the local Ethics Committee and patients and controls or their parents gave written informed consent.

**Table 1:** Baseline characteristics of study subjects.

Characteristics	Healthy controls (n = 47)	Diabetes (n = 62)	Diabetes with complications (n = 26)	p value
Age, years	25.1 ± 4.6	24.6 ± 10.4	34.8 ± 9.9*§	0.000
Male, n (%)	24 (51.1)	27 (43.5)	8 (30.8)	0.2
Smokers, n (%)	6 (12.7)	5 (8.1)	5 (19.2)	0.9
Body mass index, kg/m^2^	23.5 ± 4.2	22.8 ± 3.4	23.4 ± 2.8	0.73
Diabetes duration, years	___	10.2 ± 8.4	18.0 ± 8.0§	0.000
Hb_A1c_	5.2 ± 0.3	9.9 ± 2.6*	10.6 ± 2.6*	0.000
MUAE, μg/min	4.0 (0.5 - 17.2)	6.9 (1.6 - 20.0)*	58 (13.4 - 1608.5)*§	0.000

Hb_A1c_, glycated hemoglobin; MUAE, mean urinary albumin excretion rate.

Values represent mean ± SD or median (maximum; minimum).

* *p *< 0.05 compared to healthy subjects; § *p *< 0.05 compared to diabetes without complications.

Intravital video microscopy was carried out in the morning using a standardized and well-validated technique [4-7] in a temperature-controlled room (21-24°C). Subjects were seated and the skin of the dorsum of the middle phalanx of the left hand and proximal phalanx of the great toe was examined. Capillary density was defined as the total number of spontaneously perfused capillaries per mm^2 ^of skin. Percentage capillary recruitment was assessed by post-occlusive reactive hyperemia (PORH) after stopping arterial blood flow to the forearm and hand or to the foot by inflating a sphygmomanometer cuff over 3 min to 200 mmHg. Ten minutes after PORH, maximization of skin capillaries was obtained with 2 minutes of venous occlusion inflating the cuff to 60 mmHg (upper limb) or 90 mmHg (lower limb).

Data were analyzed by SPSS 13.0 and the Student's *t *test, Mann-Whitney, Wilcoxon and *X*^2 ^tests were used when indicated. *p *values < 0.05 were considered statistically significant.

## Results

**Figure 1:**
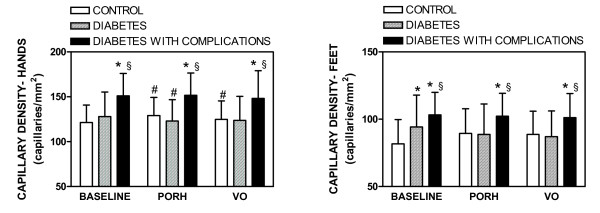
**Mean skin capillary density before (baseline) and during post-occlusive reactive hyperemia (PORH) or venous occlusion (VO) in the hands and feet of control subjects and patients with diabetes with or without vascular complications**. Values represent mean ± SD. * *p *< 0.05 compared to healthy subjects; § *p *< 0.05 compared to diabetes without complications; #*p *< 0.05 vs. baseline.

**Figure 2:**
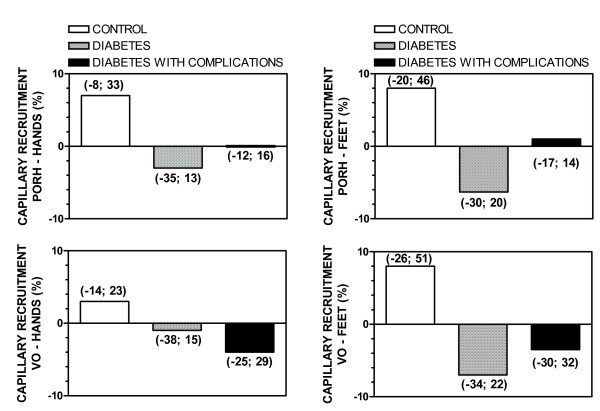
**Skin capillary recruitment during post-occlusive reactive hyperemia (PORH) or venous occlusion (VO) in the hands and feet of control subjects and patients with diabetes with or without vascular complications**. Values represent mean ± SD. * *p *< 0.05 compared to healthy subjects.

Figure 1 shows a difference in the mean capillary density (MCD) at baseline in feet, but not hands, of controls and patients without complications. Baseline MCD was significantly higher in hands and feet of patients with complications than in healthy controls or patients without complications. During PORH, MCD was also significantly higher in hands and feet of patients with complications than in controls or patients without complications. Capillary recruitment during PORH (% increase of MCD, Figure 2) was significantly higher in controls than in patients without or with complications (both in hands and feet). During venous occlusion, the MCD increase was also higher in controls than in patients without or with complications (both hands and feet). Finally, MCD during venous occlusion was also higher in patients with complications than in controls or patients without complications (both hands and feet). Within the group of diabetic patients, a positive correlation was noted between baseline MCD and age (r = 0.53; *p *< 0.001), disease duration (r = 0.30; *p *< 0.01) and female gender (r = 0.32; *p *< 0.01). The same correlations were observed concerning MCD during venous occlusion: age (r = 0.57; *p *< 0.001), disease duration (r = 0.31; *p *< 0.01) and gender (r = 0.31; *p *< 0.01). No other significant correlations were observed.

## Discussion

Microvascular disease is a major feature of type 1 diabetes that results from chronic functional and structural alterations [1]. Patients display generalized microvascular vasodilation, mainly due to altered levels of vasoactive substances, chronic plasma volume expansion and tissue hypoxia [8]. The absence of capillary recruitment in both extremities during PORH, which is related to endothelium-dependent vasodilation at the pre-capillary level, in the nutritive skin microcirculation of diabetic patients without complications has been demonstrated previously by our group [3] and was confirmed in the present study. Patients either with or without vascular complications were not able to increase capillary density during reactive hyperemia. Microvascular endothelial dysfunction associated with blunted myogenic responsiveness in type 1 diabetes may account for the pressure-induced glomerular damage in the renal pre-glomerular arterioles [9].

The new finding of the present study is that increased functional as well as structural capillary density in both extremities is observed only in diabetic patients presenting with vascular complications. Not surprisingly, capillary density was positively correlated with age and disease duration, which were significantly higher in patients with complications. Functional density corresponds to values obtained during PORH, while maximal skin capillary density (structural density) was evaluated using venous congestion, which is known to increase the red blood cell content inside capillaries and expose capillaries non-perfused at resting conditions [5]. The absence of capillary recruitment in these patients suggests that diabetic capillaries at rest are already recruited maximally as well as a loss of microvascular autoregulatory capacity. Increased capillary density at baseline (spontaneously perfused capillaries) was observed in the feet, but not in the hands of patients without complications, suggesting that microvascular alterations in type 1 diabetes initiate in the lower extremities and could contribute to complications such as chronic foot ulcers [10]. Accordingly, these microvascular alterations in lower extremities observed in our study could be considered as an early marker for foot complications. It is also worth noting that different growth factors, including vascular endothelial growth factor, are involved in the pathophysiology of diabetic kidney microvascular disease [11]. For that reason, the increased structural skin capillary density observed in our patients presenting with vascular complications could have resulted from increased levels of circulating growth factors. Taken together, our results suggest that besides microvascular vasodilation and loss of autoregulatory capacity, patients with type 1 diabetes with vascular complications present microvascular growth, probably angiogenesis, as a compensatory response to the disease process.

## Competing interests

The authors declare that they have no competing interests.

## Authors' contributions

ET and MBG conceived the study, participated in its design and coordination and wrote the manuscript. ER and ET performed the microvascular studies. RC selected and included the patients in the study. All authors read and approved the final manuscript.
